# Chest wall mechanics during pressure support ventilation

**DOI:** 10.1186/cc4867

**Published:** 2006-03-31

**Authors:** Andrea Aliverti, Eleonora Carlesso, Raffaele Dellacà, Paolo Pelosi, Davide Chiumello, Antonio Pedotti, Luciano Gattinoni

**Affiliations:** 1 Dipartimento di Bioingegneria, Politecnico di Milano, Milano, Italy; 2 Università degli Studi, Milano, Italy; 3 Dipartimento Ambiente, Salute e Sicurezza, Universita' degli Studi dell'Insubria, Varese, Italy; 4 Istituto di Anestesia e Rianimazione, Fondazione IRCCS, Ospedale Maggiore Policlinico Mangiagalli Regina Elena, Milano, Italy

## Abstract

**Introduction:**

During pressure support ventilation (PSV) a part of the breathing pattern is controlled by the patient, and synchronization of respiratory muscle action and the resulting chest wall kinematics is a valid indicator of the patient's adaptation to the ventilator. The aim of the present study was to analyze the effects of different PSV settings on ventilatory pattern, total and compartmental chest wall kinematics and dynamics, muscle pressures and work of breathing in patients with acute lung injury.

**Method:**

In nine patients four different levels of PSV (5, 10, 15 and 25 cmH_2_O) were randomly applied with the same level of positive end-expiratory pressure (10 cmH_2_O). Flow, airway opening, and oesophageal and gastric pressures were measured, and volume variations for the entire chest wall, the ribcage and abdominal compartments were recorded by opto-electronic plethysmography. The pressure and the work generated by the diaphragm, rib cage and abdominal muscles were determined using dynamic pressure-volume loops in the various phases of each respiratory cycle: pre-triggering, post-triggering with the patient's effort combining with the action of the ventilator, pressurization and expiration. The complete breathing pattern was measured and correlated with chest wall kinematics and dynamics.

**Results:**

At the various levels of pressure support applied, minute ventilation was constant, with large variations in breathing frequency/ tidal volume ratio. At pressure support levels below 15 cmH_2_O the following increased: the pressure developed by the inspiratory muscles, the contribution of the rib cage compartment to the total tidal volume, the phase shift between rib cage and abdominal compartments, the post-inspiratory action of the inspiratory rib cage muscles, and the expiratory muscle activity.

**Conclusion:**

During PSV, the ventilatory pattern is very different at different levels of pressure support; in patients with acute lung injury pressure support greater than 10 cmH_2_O permits homogeneous recruitment of respiratory muscles, with resulting synchronous thoraco-abdominal expansion.

## Introduction

In intensive care pressure support ventilation (PSV), a form of assisted mechanical ventilation, is among the modes most commonly employed to decrease the patient's work of breathing without neuromuscular blockade [1]. It is known that for optimal unloading of the respiratory muscles, the ventilator should cycle in synchrony with the activity of the patient's respiratory rhythm. Patient-ventilator asynchrony frequently occurs at various levels of PSV. The interplay between the respiratory muscle pump and mechanical ventilator is complex, and problems can arise at several points in the respiratory cycle. Ventilators may not be in synchrony with the onset of the patient's inspiratory effort (for instance inspiratory asynchrony, or trigger asynchrony). In addition, patient-ventilator asynchrony may be present during the onset of exhalation (for instance expiratory asynchrony). Both inspiratory and expiratory asynchrony cause discomfort and unnecessary increased work of breathing, and are associated with difficult weaning from mechanical ventilation.

Synchronization of respiratory muscle action and the resulting chest wall kinematics (rib cage and abdominal motion) are therefore generally considered valid indicators of the patient's adaptation to the ventilator [2,3]. However, most information related to the interaction between patient and ventilator during PSV was obtained in mechanically ventilated patients suffering an exacerbation of chronic obstructive pulmonary disease (COPD) [4,5]. In contrast, little information is available on non-COPD patients with moderate-to-severe respiratory failure. Moreover, the devices that are commonly used to assess chest wall kinematics are only able to provide a qualitative description of asynchrony and/or paradoxical motion. The technique of opto-electronic plethysmography (OEP) [6-8] allows one to obtain accurate measurements of changes in volume for the total chest wall and its compartments (rib cage and abdomen) in mechanically ventilated patients. Combining these volumes with oesophageal and gastric pressure measurements, it is possible to assess the action of the respiratory muscles and chest wall dynamics, facilitating better understanding of the patient-ventilator interaction.

The aim of the present study was to investigate the effects of different levels of PSV on the ventilatory pattern and the action of the different respiratory muscle groups (such as inspiratory rib cage muscles, diaphragm and expiratory abdominal muscles) in a group of non-COPD patients with severe-to-moderate respiratory failure.

## Method

### Participants

We studied nine patients with acute lung injury/acute respiratory distress syndrome, who were ventilated with a Siemens Servo 900C (Siemens-Elema, Solna, Sweden) and were considered able to tolerate low level PSV (Table 1). Exclusion criteria included age below 16 years, haemodynamic instability and history of COPD. The study was approved by the institutional review board of the hospital, and informed consent was obtained in accordance with national regulations.

### Protocol

At the start of the study, PSV was instituted with pressure support at 10 cmH_2_O, positive end-expiratory pressure at 10 cmH_2_O, oxygen fraction as clinically indicated (Table 1) and trigger sensitivity at 0.5 cmH_2_O. The patients were then ventilated with three different levels of pressure support (5, 15 and 25 cmH_2_O) and with positive end-expiratory pressure at 10 cmH_2_O. Each step was randomized and maintained for about 15 minutes. Data were recorded during the last 3 minutes of each step and, in two patients, during the transitions between two different levels of pressure support.

Flow was measured using a heated pneumotachograph (HR 4700-A; Hans Rudolph, Kansas, MO, USA) and a differential pressure transducer (MP-45; Validyne, Northridge, CA, USA). Airway opening pressure was measured by a piezoresistive transducer (SCX01; Sensym, Milpitas, CA, USA). Oesophageal (Pes) and gastric (Pga) pressures were measured using standard latex balloon-tipped catheters (Bicore, Irvine, CA, USA), which were inflated with 0.5–1 and 1–1.5 ml air, respectively, and connected to similar pressure transducers (SCX05; Sensym). The position and validity of the pressure signals were assessed using chest radiography and the occlusion test [9].

Blood gas analysis was performed at the end of each pressure support step (IL1620; Instrumentation Laboratory, Lexington, MA, USA). The level of sedation was evaluated using the Ramsey scale [10].

The chest wall volume (Vcw) and the volumes of its compartments were measured using OEP (OEP System, BTS, Milano, Italy), as previously described in detail [6-8]. Forty-five reflecting markers (composed of plastic hemispheres of 6 mm diameter covered by a thin film of retroreflective paper) were placed over the chest wall from clavicles to pubis and secured using biadhesive hypoallergenic tape. Each marker was tracked using four video cameras, positioned about 2 m above the patient and inclined downward, and the three-dimensional position of each marker was reconstructed by stereo-photogrammetry at a sampling rate of 50 Hz. For volume computation, the chest wall surface was approximated by 182 triangles connecting the markers. Then, using Gauss' theorem, the Vcw and the volumes of its compartments were calculated. We assumed a three-compartment model of the chest wall, as originally proposed by Ward and coworkers [11] and Aliverti and colleagues [12]; this model comprises pulmonary rib cage, abdominal rib cage and abdomen. The pulmonary rib cage was defined as extending caudally from the markers placed on the clavicular line to those placed at the xiphoid level, assumed to be the cephalic extremity of the area of apposition of the diaphragm at functional residual capacity. The abdominal rib cage was defined as extending from the xiphoid level to the lower costal margin. Finally, the abdomen was defined as extending from the lower costal margin to the anterior superior iliac crest line [6,7]. The volumes of the compartment were summed to yield the Vcw: Vcw = Vrc, p + Vrc, a + Vab = Vrc + Vab (where Vrc, p is the pulmonary rib cage volume, Vrc, a is the abdominal rib cage volume, Vab is the abdominal volume, and Vrc is the volume of the entire rib cage).

### Data analysis

In each patient, the volumes, flow and pressure tracings were normalized with respect to time in order to derive ensemble averages over all breaths and to derive an 'average' respiratory cycle at each level of pressure support. This was done by analyzing all breaths during the recording period (3 minutes for each step in each patients); normalizing each breath with respect to time by re-sampling data (with linear interpolation) to obtain a fixed number of samples (*n *= 100) between two consecutive onsets of inspiratory effort; and computing the ensemble averages for Vrc, p, Vrc, a, Vab, Vcw, flow, Pes, gastric pressure and transdiaphragmatic pressure (Pdi) for each patient at each level of pressure support and expressing them as percentage of total respiratory cycle time.

**Figure 1:**
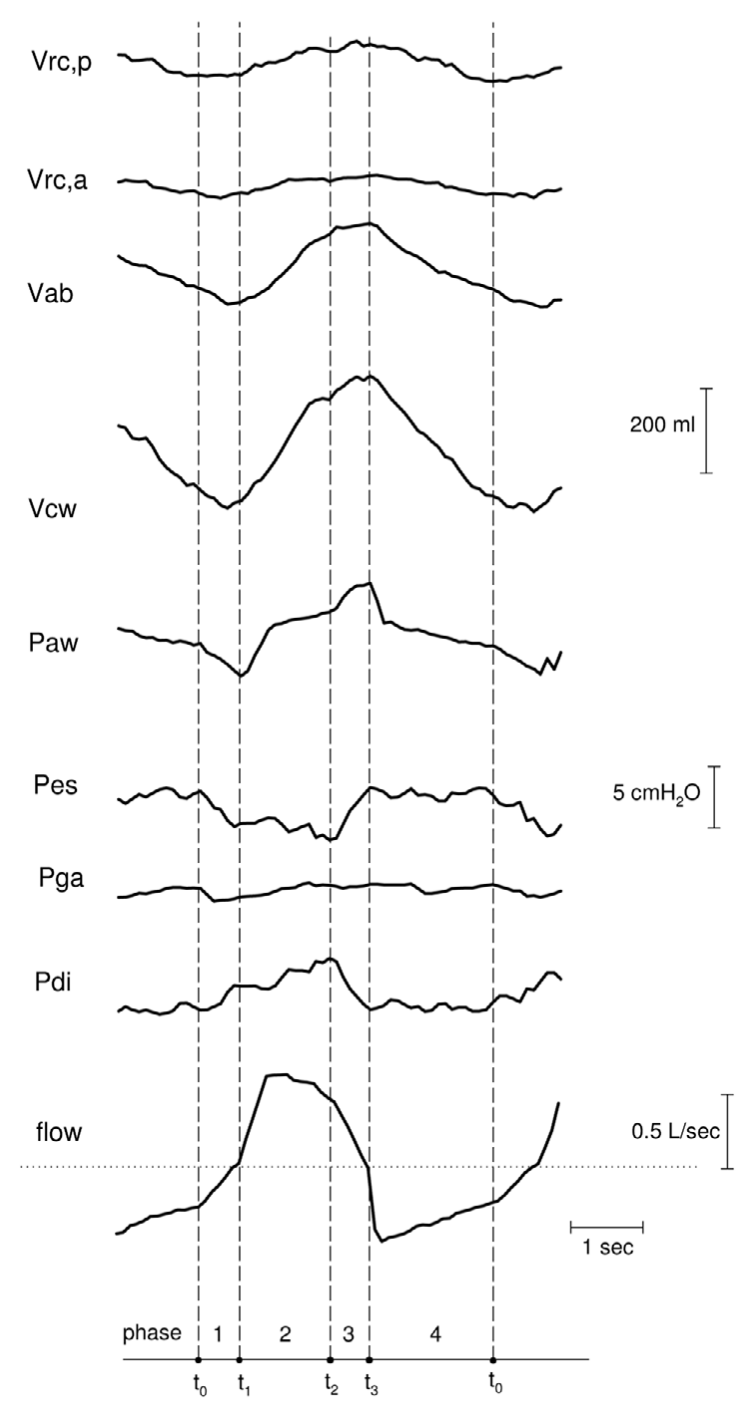
Experimental tracings obtained during a breath from patient receiving PSV (pressure support 5 cmH_2_O). Time t_0 _is defined as where Pes starts to decrease; t_1 _is the onset of inspiratory flow; t_2 _is where Pes starts to increase; and t_3 _is the end of inspiration. Vab, abdominal volume; Vcw, chest wall volume; Vrc, a, volume of the abdominal rib cage; Vrc, p, volume of the pulmonary rib cage; Paw, airway pressure; Pdi, transdiaphragmatic pressure; Pes, oesophageal pressure; Pga, gastric pressure.

In each respiratory cycle four times (t) and phases were identified (Figure 1): phase 1 was defined as extending from t_0 _(when Pes begins to fall) to t_1 _(the beginning of inspiratory flow); phase 2 was from t_1 _to t_2 _(when Pes begins to increase); phase 3, with Pes continuously rising, was from t_2 _to t_3 _(the end of inspiration); and phase 4 was from t_3 _to t_4 _(expiration).

### Estimation of muscle pressure and work

Vcw was plotted against Pes with pressure support at 25 cmH_2_O, and we assumed that the obtained pressure-volume curve of the chest wall represented the relaxation curve of the system [13]. Indeed, the pressure developed by the respiratory muscles (Pmus) was measured as the distance along the pressure axis between the dynamic Vcw-Pes loop and this relaxation curve.

The pressure developed by the diaphragm was estimated by transdiaphragmatic pressure (Pdi), computed as Pga-Pes.

Similarly to the Pmus, the pressure developed by rib cage muscles (Prcm) was measured as the distance along the pressure axis between the dynamic Vrc, p-Pes loop and the relaxation curve of the pulmonary rib cage. As reported previously [12,14], estimation of Prcm requires use of Vrc, p rather than Vrc, based on the assumption that the lung-apposed part of the rib cage is the only part of the rib cage subjected to pleural pressure and the action of the inspiratory rib cage muscles.

The pressure developed by the abdominal muscles was measured as the distance along the pressure axis between the dynamic Vab-abdominal pressure loop and the relaxation curve of abdomen (Vab versus Pga with pressure support set at 25 cmH_2_O).

Displacements of dynamic pressure volume curves upward and to the left of the relaxation curves, measured with pressure support at 5, 10 and 15 cmH_2_O, were taken as evidence of inspiratory muscle mechanical activity. Displacements downward and to the right were taken as evidence of expiratory muscle activity [15,16].

Integrating the area between inspiratory Pes-Vcw tracings with pressure support at 5, 10 and 15 cmH_2_O, and the curve at 25 cmH_2_O during phases 1, 2 and 3 (defined above) provided the total inspiratory work of breathing (WOB). Muscle pressures and WOB were derived considering the ensemble averages of the breaths recorded during each run.

The pressure-time product per minute was calculated as the integral of the Pes tracing versus time from the beginning of the inspiratory deflection to the end of the inspiratory flow and multiplied by the respiratory rate [17]. Occlusion pressure (P_0.1_) was calculated as the Paw drop over the initial 100 ms of inspiratory effort during occlusion manoeuvres [18]. Asynchrony between rib cage and abdominal motion was assessed by calculating the phase angle between Vab and Vrc loop with the method decribed by Bloch and coworkers [19].

### Statistical analysis

To study the effect of the different pressure support levels on the different variables, we applied a one-way analysis of variance on repeated measures. A *post hoc *Bonferroni test was applied to verify the statistical significance of the differences between all pairs of means. *P *< 0.05 was considered statistically significant. All data are expressed as mean ± standard error of the mean.

## Results

### Overall ventilatory pattern

**Figure 2:**
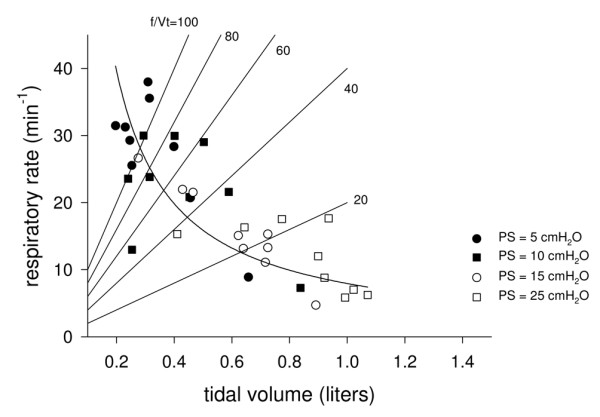
Relationship between V_t _and respiratory rate. Shown is the relationship between V_t _and respiratory rate (f) in the patients at different levels of pressure support: 5 cmH_2_O (closed circles), 10 cmH_2_O (open circles), 15 cmH_2_O (closed squares) and 25 cmH_2_O (open squares). The straight lines represent isopleths of different values of f/V_t _(20, 40, 60, 80 and 100 l^-1^·min^-1^). The curved line is the fitting of data points by the following equation: f = K/V_t _(where K = 7.9385 ± 0.4324). PS, pressure support; Vt, tidal volume.

As shown in Table 2, total minute ventilation was unmodified by varying the pressure support from 5 to 25 cmH_2_O because of decreased respiratory rate and increased tidal volume when pressure support increased. The resulting gas exchange was also unmodified. Interestingly, as shown in Figure 2, with pressure support at 5 cmH_2_O most patients exhibited a frequency/tidal volume ratio (f/Vt) index greater than 100 (rapid shallow breathing), which progressively and slowly decreased when the pressure support was increased to 10, 15 and 25 cmH_2_O (Table 2).

### Duration of the breathing phases

**Figure 3:**
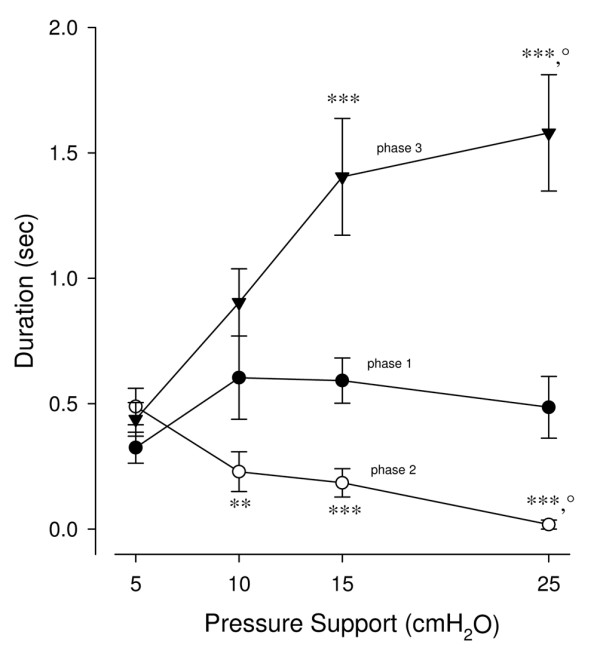
Relationships between pressure support levels and duration of the various phases of inspiration. The phases (phase 1 [closed circles], phase 2 [open circles] and phase 3 [closed triangles]) are defined in the text. Data are expressed as mean ± standard error of the mean. **P < 0.01, ***P < 0.001, versus pressure support = 5 cmH_2_O. °*P *< 0.05, versus pressure support = 10 cmH_2_O.

As shown in Figure 3, the duration of phase 1 was independent of the pressure support level. However, the duration of phase 2 (in which the patient's effort is greater than the action of the ventilator) was strongly related to pressure support, being progressively shorter with increasing pressure support. As phase 2 shortened, the duration of phase 3 (in which the action of the ventilator is greater than the inspiratory effort made by the patient) progressively increased with increasing pressure support from 5 to 25 cmH_2_O. Phase 4 (expiration) behaved similarly to phase 3.

The increase in inspiratory time (the sum of phases 2 and 3) with increasing pressure support was less than the increase in expiratory time (the sum of phases 1 and 4). Thus, most of the decrease in frequency was due to the increased expiratory time.

The inspired volume during phases 2 and 3 was associated with the duration of these phases and progressively increased from pressure support 5 cmH_2_O to 15 cmH_2_O. Consequently, the mean inspiratory flow (ΔV/ [duration of phases 2 and 3]) was almost constant at pressure support 5, 10 and 15 cmH_2_O (0.411 ± 0.035 l/s, 0.462 ± 0.058 l/s and 0.430 ± 0.051 l/s, respectively) and it increased significantly only at pressure support 25 cmH_2_O (0.631 ± 0.061 l/s; *P *< 0.001).

### Pressures developed by respiratory muscles at different phases

**Figure 4:**
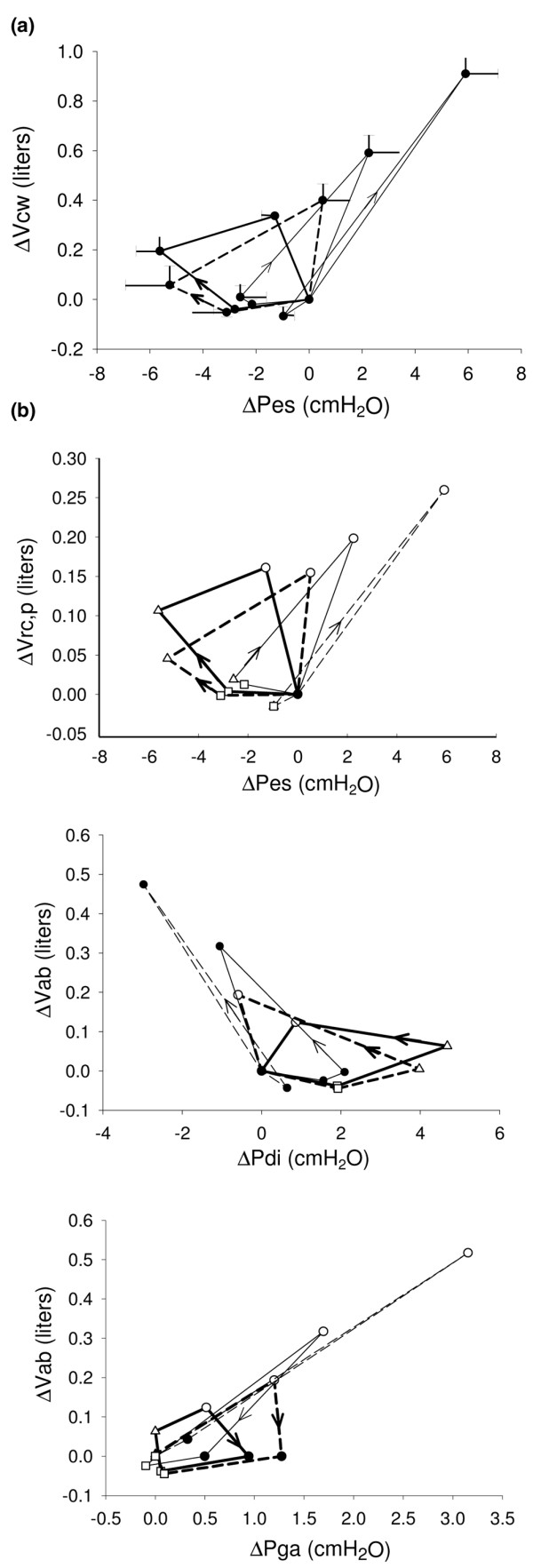
Pressure-volume dynamic relationship of the total and compartment chest wall. **(a) **Change in oesophageal pressure (ΔPes) versus chest wall volume changes (ΔVcw).  **(b) **Upper panel: changes in oesophageal pressure (ΔPes) versus pulmonary rib cage volume changes (ΔVrc, p); averaged loops. Middle panel: changes in transdiaphragmatic pressure (ΔPdi) versus abdominal volume changes (ΔVab); averaged loops. Lower panel: changes in gastric pressure (ΔPga) versus abdominal volume changes (ΔVab); Each point represents the mean ± standard error of the mean (i.e. the average of all patients at the different times [see definition in Figure 1]): The loops refer to the different levels of pressure support: 5 cmH_2_O (solid thick line), 10 cmH_2_O (dased thick line), 15 cmH_2_O (solid thin line) and 25 cmH_2_O (dashed thin line). The arrows indicate the direction of the loops. The symbols in (b) are  t_0 _(closed circles), t_1 _(open squares), t_2 _(open triangles) and t_3 _(open circles).

Figure 4a summarizes the average behaviour of the dynamic pressure-volume curve of the total chest wall (Pes-Vcw) at the different pressure support levels, split into the different phases, whereas Figure 4b shows partitioning into rib cage and diaphragm-abdominal compartments (for instance Vrc, p-Pes, Vab-Pdi and Vab-Pga relationships). In these figures, the starting volumes and pressures (for instance the volumes and pressures at the onset of the inspiratory effort at the beginning of phase 1) were considered zero.

#### Total chest wall volume-pressure dynamic loops

As shown in Figure 4a, during phase 1 the total chest wall volume slightly decreased, and the pressure generated by the patient to trigger the ventilator ranged between 1 and 3 cmH_2_O at the different levels of pressure support. During phase 2 (during which the patient continued to contribute effort) the total muscle pressure generated by the patient (for instance the horizontal distance between each point and the corresponding pressure on the expiratory limb of the loop at 25 cmH_2_O) was higher at pressure support 5 cmH_2_O and decreased at 10 and 15 cmH_2_O. At the end of phase 3 the pressure developed by the inspiratory muscles was still higher with pressure support at 5 cmH_2_O than at 10 and 15 cmH_2_O. Being the points at the end of phase 3 to the left of the relaxation line, these results indicate residual contraction of the inspiratory muscles at the beginning of expiration and a gradual relaxation during expiration. These data indicate the following: the pressure developed by the inspiratory muscles to trigger the ventilator is independent of the pressure support level; the total pressure developed by the inspiratory muscles during phase 2 increases with decreasing pressure support level; and at the beginning of expiration (open circles in Figure 4a) there is persistent inspiratory action of the inspiratory muscles, which is present throughout expiration. This behaviour is associated with increased WOB in the form of negative work.

It is worth noting, however, that the inter-patient variability was considerable. In fact, in two out of nine patients, with pressure support at 5 cmH_2_O the pressure at the end of inspiration was slightly higher than the corresponding pressure on the relaxation curve, indicating net expiratory muscle mechanical activity.

#### Compartmental (rib cage, diaphragm and abdomen) volume-pressure dynamic loops

In Figure 4b (upper panel) the Vrc, p-pleural pressure loops are shown as an expression of the action of the inspiratory rib cage muscles. The behaviour of this compartment was similar to that of the total chest wall.

In Figure 4b (middle panel) the Vab-Pdi loops are shown as an expression of the action of the diaphragm. Pdi at the end of phase 1 was independent of the pressure support level. At the end of phase 2, Pdi decreased with increase in pressure support. In contrast to the pulmonary rib cage compartment, at the end of phase 3 the points were very close to the relaxation line, indicating lesser persistent inspiratory action of the diaphragm at the onset of expiration.

In Figure 4b (lower panel), the Vab-Pga loops are shown as an expression of the action of the expiratory abdominal muscles. At all pressure support levels, Pga decreased during phase 1, did not change during phase 2 and increased during phase 3. During phase 4, at low levels of pressure support (5 and 10 cmH_2_O) the dynamic loops deviated from the relaxation line, indicating expiratory action of the abdominal muscles (increasing Pga with decreasing Vab).

#### Compartmental chest wall volume changes

**Figure 5:**
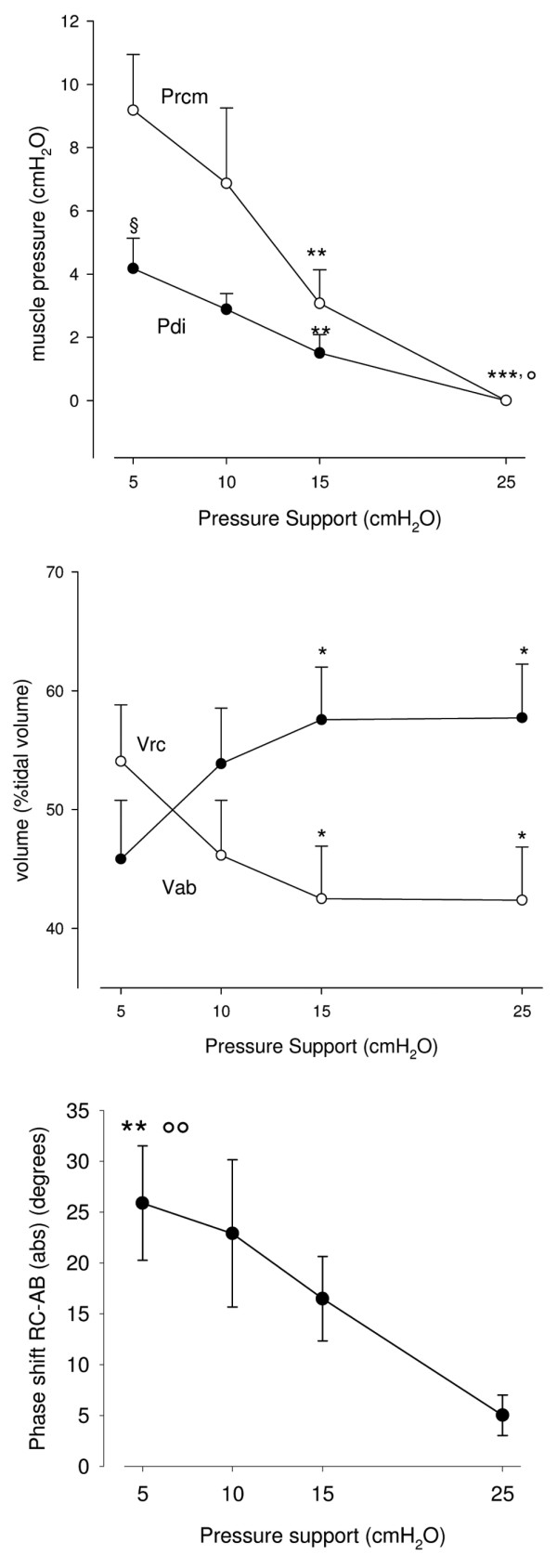
Relationship between pressure support level and muscle pressure, chest wall volume distribution and synchronization. **(a) **Mean ± standard error of the mean (SEM) values of transdiaphragmatic pressure (Pdi; closed symbols) and rib cage muscle pressure (Prcm; open symbols) at different levels of pressure support. **P < 0.01,***P < 0.001, versus pressure support at 5 cmH_2_O. °*P *< 0.01, versus pressure support at 10 cmH_2_O. *P *< 0.05, versus Prcm. **(b) **Mean ± SEM values of percentage contribution to tidal volume of abdomen (Vab; closed symbols) and rib cage (Vrc; open symbols) at different levels of pressure support. *P < 0.05, versus Pressure support at 5 cmH_2_O. **(c) **Mean ± SEM values of absolute values of phase shift between rib cage (RC) and abdomen (AB) at different levels of pressure support. **P < 0.01, versus pressure support at 25 cmH_2_O. °°*P *< 0.01 versus pressure support at 15 cmH_2_O.

As shown in Figure 5 (upper panel), with increasing pressure support peak values of Prcm (measured at the end of phase 2) were consistently higher than peak values of Pdi (*P *< 0.001). with pressure support at 5 cmH_2_O, the ratio between Prcm and Pdi was significantly higher than at other levels of pressure support.

Accordingly, the distribution of the inspired tidal volume in the different chest wall compartments was dependent on the different levels of pressure support (Figure 5, middle panel). The abdomen expanded more with pressure support at 15 and 25 cmH_2_O than it did with pressure support at 5 and 10 cmH_2_O (*P *< 0.05).

The phase shifts between rib cage and abdominal volume variations with pressure support at 5 cmH_2_O were similar to those with pressure support at 10 cmH_2_O, but they were significantly higher than with pressure support at 15 and 25 cmH_2_O (Figure 5, lower panel).

## Discussion

In this study, conducted in a group of mechanically ventilated non-COPD patients with severe-to-moderate respiratory failure, we found that respiratory rate and tidal volume changes were good bedside indicators of WOB and respiratory drive. Furthermore, pressure support levels below 15 cmH_2_O increased the following: the pressure developed by the inspiratory muscles, and the contribution of rib cage compartment to the total tidal volume; the simultaneous post-inspiratory action of the rib cage muscles and expiratory action of the abdominal muscles; and the phase shift between rib cage and abdominal compartments.

### Ventilatory pattern, gas exchange and respiratory effort

The pattern of breathing was modified markedly by increasing the level of pressure support, with increased tidal volume and reductions in respiratory rate, WOB and P_0.1_. Furthermore, our data indicate that arterial carbon dioxide tension, minute ventilation and inspiratory flow were maintained nearly constant, independent of pressure support level. This suggests that there are different mechanisms of adaptation resulting in different breathing patterns at different levels of pressure support, from a pattern similar to rapid shallow breathing (pressure support at 5 cmH_2_O) to one similar to completely passive pressure control ventilation (pressure support at 25 cmH_2_O). The relationship we found between respiratory rate and tidal volume, for different f/Vt isopleths, was similar to that reported by Yang and Tobin [20] in spontaneously breathing individuals. Few previous studies have systematically investigated the effects of pressure support level on breathing pattern in non-COPD patients with respiratory failure. Tokioka and coworkers [21] assessed the effect of pressure support on breathing pattern and WOB in 10 postoperative patients. They found no significant changes in minute ventilation between pressure support at 5 and 10 cmH_2_O. Van de Graaf and coworkers [22] evaluated 33 patients who undergone aorto-coronary bypass with pressure support ranging from 0 to 30 cmH_2_O. They found no change in minute ventilation, arterial carbon dioxide tension, or pH despite large changes in both rate and depth of breathing. They also found a marked reduction in WOB with increasing pressure support levels. In 10 patients with acute respiratory failure, Alberti and colleagues [18] found a reduction in respiratory rate and WOB and an increase in tidal volume, with unchanged minute ventilation, mainly at higher levels of pressure support. Furthermore, they found a good correlation between P_0.1 _and WOB.

In postoperative septic patients, Perrigault and coworkers [23] found that the minute ventilation and breathing pattern parameters were unaffected by the level of pressure support, and P_0.1 _was more useful for setting the optimal level of respiratory assistance. In a more recent study, Chiumello and coworkers [24], in their evaluation of 10 patients with acute respiratory failure, found an increase in tidal volume and reductions in respiratory rate, WOB and P_0.1_, with minute ventilation and arterial carbon dioxide tension unchanged, with pressure support increasing from 5 to 15 cmH_2_O.

The relationship we found between the respiratory rate and tidal volume, for different f/Vt isopleths, was similar to that reported by Yang and Tobin [20] in spontaneously breathing individuals during weaning from mechanical ventilation. Our data suggest that respiratory rate and tidal volume changes are good bedside indicators of WOB and respiratory drive, and therefore we believe that f/Vt may be considered an indicator of adequacy of pressure support level.

However, to our knowledge, no data are available on partitioning of the WOB into the contributions made by the different respiratory muscle groups at different pressure support levels in mechanically ventilated patients. OEP, which was initially developed to study chest wall mechanics in healthy individuals in erect and seated positions [12,25], was recently introduced into the intensive care unit setting (supine [6-8] and prone [7] positions). In previous studies the method was validated in patients during both PSV and continuous positive pressure ventilation. The accuracy of the method in this setting was assessed by comparing OEP with spirometry and pneumotachography, and was found to be +1.7 ± 5.9% and -1.6 ± 5.4%, respectively [6]. Indeed, we believe that this method is suitable for volume recordings in the intensive care unit; advantages in this setting would include the possibility to partition the chest wall, absence of drift and the noninvasive nature of the technique.

### Analysis of breathing phases

In order to describe these phenomena in detail, we chose to partition the respiratory cycle into different phases. Traditionally, the respiratory cycle is divided into three frames [2]: the ventilator trigger, the pressurization phase and the expiration phase. As was also recently suggested by Tassaux and coworkers [4], we opted to split the pressurization period into two phases (Figure 1), because we believe that they correspond more precisely to the underlying physiological and mechanical phenomena (predominant patient or ventilator effort). Indeed, we consider patient activity to be predominant when the oesophageal pressure decreased during pressurization, and the ventilator activity predominant when the oesophageal pressure rose.

#### Phase 1

In these patients the unassisted breathing effort should mainly reflect two phenomena, namely the neurological drive and the interaction between the inspiratory muscles and the mechanical characteristics of the inspiratory valve, because the intrinsic positive end-expiratory pressure was nil. Although the triggering pressure is independent of the drive, the time to achieve the triggering pressure is an index of drive. The phase 1 data suggest that the neurological drive is greater at a pressure support of 5 cmH_2_O. In fact, ΔPes/Δt was significantly greater at a pressure support of 5 than at 25 cmH_2_O (-7.9 ± 2.9 versus -1.8 ± 1.0; *P *< 0.01). Moreover, at a pressure support of 5 cmH_2_O the P_0.1 _was tenfold that at a pressure support of 25 cmH_2_O, and it progressively decreased in the intermediate stages (pressure support 10 and 15 cmH_2_O). The patients presumably maintained their arterial carbon dioxide and minute ventilation constant by increasing neurological drive in response to the low pressure support. This was achieved by recruiting both inspiratory rib cage muscles and the diaphragm independent of the level of pressure support.

#### Phase 2

In this phase the patient's effort is greater than the action of the ventilator because Pes continuously decreases. The level of pressure support has a potent influence on this phase [2]. In fact, at a pressure support of 25 cmH_2_O the duration of this phase was nil. At pressure support levels lower than 25 cmH_2_O it progressively increased until it reached 0.48 ± 0.08 s at a pressure support of 5 cmH_2_O. The decrease in Pes was also directly related to the Pmus (the pressure developed by the respiratory muscles) applied by the patient and inversely related with the level of pressure support. Interestingly, at a pressure support of 25 cmH_2_O there was no phase 2 and Pes increased as soon as the inspiratory valve opened. Very little (if any) inspiratory effort was made by the patient, suggesting near complete relaxation.

The values of pressures reached at the end of this phase suggested that the action of the inspiratory rib cage muscles, as compared with that of the diaphragm, progressively decreased at higher rates with increasing level of pressure support. This was associated with a different chest wall configuration at end-inspiration, with abdominal compartment volume being greater at higher levels of pressure support.

#### Phase 3

In this phase the pressure supplied by the ventilator was greater than the patient's effort because Pes rose. At a pressure support of 5 cmH_2_O the duration of this phase was significantly shorter because most of the volume was already delivered in phase 2, whereas at pressure support levels of 10, 15 and 25 cmH_2_O the duration became progressively greater.

More interestingly, we found increased inspiratory tone activity at the end of phase 3, just at the beginning of expiration. This suggest post-inspiratory activity of the diaphragm and rib cage muscles, which was previously reported both in normal spontaneously breathing individuals [26], in anaesthetized normal individuals [27] and in anaesthetized khyphoscoliotic patients [28].

Inspiratory muscle activity during expiration (work done while muscles are lengthened) involves negative work and energy expenditure. Behrakis and coworkers [27] reported that 36–74% of the elastic energy stored during inspiration may be wasted in terms of negative inspiratory muscle work in anaesthetized, spontaneously breathing normal individuals. However, this may also have some advantages such as preventing the lungs from emptying too rapidly, which may affect gas exchange adversely [28].

#### Phase 4

Our data suggest the presence of expiratory abdominal muscle action at pressure support levels 5 and 10 cmH_2_O. Expiratory activity was previously reported in mechanically ventilated patients with COPD during PSV [29]. This was extremely variable and occurred either in the last phase of inspiration or only during exhalation [30]. We were unable to find any previous data on expiratory muscle activity at different levels of pressure support in patients with acute respiratory failure. Nevertheless, our data indicate that the simultaneous presence of post-inspiratory action of the inspiratory rib cage muscles and the action of expiratory abdominal muscles lead to asynchronous motion of the chest wall (for instance an increasing phase shift between rib cage and abdomen) with decreasing levels of pressure support.

Our data also suggest that, in patients with acute respiratory failure, levels of pressure support lower than 15 cmH_2_O increase the action of respiratory rib cage muscles relative to the diaphragm, resulting in predominant distribution of tidal volume into the rib cage compartment. Furthermore, we observed an increased post-inspiratory action of the inspiratory muscles at the beginning of expiration. This pattern of recruitment of inspiratory and expiratory muscles finally resulted in asynchronous thoraco-abdominal displacement at levels of pressure support lower than 15 cmH_2_O.

## Conclusion

In patients with severe-to-moderate respiratory failure, the level of pressure support had an impact on the pattern of respiratory muscle recruitment. In particular, when the level of pressure support was lower or equal to 10 cmH_2_O, inspiratory rib cage muscles were invariantly active during triggering, post-triggering and expiration, whereas expiratory muscles were recruited during expiration. Thus, pressure support greater than that 10 cmH_2_O is necessary in patients with acute lung injury to allow homogeneous recruitment of the respiratory muscles, with resulting synchronous thoraco-abdominal expansion.

## Key messages

• 	PSV should not be considered a 'unique form' of ventilation, because its effects may be quite different depending on the pressure support level.

• 	At the different levels of pressure support minute ventilation was maintained constant, with large variations in breathing frequency/tidal volume ratio.

• 	Pressure support levels lower than 15 cmH_2_O increase the following: the pressure developed by the inspiratory muscles, and the contribution of the rib cage compartment to the total tidal volume; the simultaneous post-inspiratory action of the rib cage muscles and expiratory action of the abdominal muscles; and the phase shift between rib cage and abdominal compartments.

• 	Pressure support levels greater than 10 cmH_2_O are necessary to allow homogeneous recruitment of respiratory muscles, with resulting synchronous thoraco-abdominal expansion.

## Abbreviations

COPD = chronic obstructive pulmonary disease; f/Vt = frequency/tidal volume ratio; OEP = opto-electronic plethysmography; P_0.1 _= occlusion pressure; Pdi = transdiaphragmatic pressure; Pes = esophageal pressure; Pga = gastric pressure; Pmus = pressure developed by the respiratory muscles; Prcm = pressure developed by rib cage muscles; PSV = pressure support ventilation; Vab = abdominal volume; Vcw = chest wall volume; Vrc = rib cage volume; Vrc, a = abdominal rib cage volume; Vrc, p = pulmonary rib cage volume; WOB = work of breathing.

## Competing interests

Politecnico of Milano University (Institution of AA, RD and AP) owns patents on OEP, which were licensed to BTS spa company. EC, PP, DC and LG do not have financial relationships with commercial entities that have an interest in the subject of this manuscript.

## Authors' contributions

AA, RD, PP, EC and DC performed the study and carried out data collection. AA, PP and LG drafted the manuscript. AA and EC performed the statistical analysis. AA, PP, RD, AP and LG conceived the study and participated in its design and coordination. All authors read and approved the final manuscript.

## Figures and Tables

**Table 1:** Patient characteristics

Patient	Sex	Age (years)	BMI (kg/m^2^)	PaO_2_/FiO_2_	Fio_2_	PEEP	Diagnosis	Study day
1	F	69	29.14	230.0	0.40	6	Chemical poisoning	28
2	F	74	29.38	240.0	0.35	15	Pneumonia	12
3	F	60	27.55	293.3	0.30	3	Septic shock	38
4	M	49	24.69	380.0	0.40	2	Septic shock in polytrauma	79
5	M	67	22.86	280.0	0.40	8	Haemorragic shock	29
6	M	65	31.25	237.1	0.35	2	Post-anoxic coma	9
7	M	47	30.47	274.3	0.40	4	Polytrauma	50
8	F	34	22.04	410.0	0.30	11	Pneumonia	5
9	M	69	27.78	153.3	0.45	11	Septic shock	37
Mean		59.3	27.2	277.6	0.37	6.9		31.9
SD		13.2	3.3	78.3	0.1	4.6		23.1

BMI, body mass index; F, female; FiO_2_, fraction of inspired oxygen; M, male; PEEP, positive end-expiratory pressure.

**Table 2:** Ventilatory pattern, gas exchange and respiratory effort

Parameter	*P*	Pressure support (cmH_2_O)			
		
		5	10	15	25
Tidal volume (l)	<0.001	0.340 ± 0.048	0.432 ± 0.064	0.610 ± 0.063	0.852 ± 0.070
Frequency (minute^-1^)	<0.001	27.7 ± 2.9	22.1 ± 2.6	15.9 ± 2.2	11.9 ± 1.7
Minute ventilation (l/minute)	NS	8.5 ± 0.8	8.9 ± 1.2	8.6 ± 0.7	9.5 ± 1.2
Pao_2_/Fio_2 _(mmHg)	0.042	299.3 ± 22.0	263.1 ± 22.8	288.2 ± 22.3	320.0 ± 25.4
Paco_2 _(mmHg)	NS	35.4 ± 2.4	33.9 ± 2.7	33.9 ± 2.2	33.8 ± 2.0
f/Vt (1/l·minute)	<0.001	97.9 ± 15.4	61.2 ± 9.9	33.3 ± 9.3	16.2 ± 3.6
WOB (J/minute)	<0.001	4.9 ± 1.0	3.8 ± 1.7	1.2 ± 0.6	0.0 ± 0.0^a^
PTP (cmH_2_O s/minute)	<0.001	106.1 ± 18.3	61.9 ± 21.8	16.3 ± 8.6	0.1 ± 0.1
P_0.1 _(cmH_2_O)	<0.001	2.0 ± 0.5	1.5 ± 0.3	0.9 ± 0.2	0.3 ± 0.2

Where applicable, values are expressed as mean ± standard error of the mean. ^a^Zero work of breathing (WOB) is the consequence of our assumption that, at 25 cmH_2_O, the respiratory system is in a fully relaxed state. *P *values refer to one-way analysis of variance on repeated measures (for different levels of pressure support). FiO_2_, fraction of inspired oxygen; f/Vt, frequency/tidal volume ratio; NS, not significant; P_0.1_, occlusion pressure; PaCO_2_, arterial carbon dioxide tension; PaO_2_, arterial oxygen tension; PTP, pressure time product.
